# Supplementary material for: Deciphering the Autoantibody Response to the OJ Antigenic Complex
Source: Diagnostics (Basel). 2023 Jan 3;13(1):156. doi: 10.3390/diagnostics13010156 (PMC9818932; doi:10.3390/diagnostics13010156)

Supplementary Materials

Supplemental Table S1: Cohort of anti-OJ positive samples (n=39) detected by line immunoassay (LIA)*

Sample No.	Sample ID	cN-1A	Ro52	**OJ**	EJ	PL-12	PL-7	SRP	Jo-1	PM75	PM100	Ku	SAE1	NXP2	MDA5	TIF1 γ	Mi-2 β	Mi-2 α
1	X294			14				11										
2	X343		48	65							13							
3	X271			15														
4	X379			11														
5	X709			42												90		
6	X410			13														
7	X299			18		11												
8	X446			25														
9	X794			23														
10	X566		21	12						15						14		
11	X856			11														
12	X331			48														
13	X669			14								17						
14	X286			32														
15	X648			37														
16	X627			20														
17	X391	48		13			34					25						
18	X370			11														
19	X325			12						15								
20	X870			15														
21	X967			16														
22	X873			13														
23	X044			51														
24	X393			29								10						
25	X657			86														
26	X141			18														
27	A382			12														
28	X003			17														
29	X671			26				23										
30	X757		82	18								10		110				
31	X291			11								11						
32	X063			16														
33	X029	45		12														
34	X556			30														
35	X077			18														
36	X007			11														
37	X061			11														
38	X001			43														
39	X002			15														

* **LIA:** Results obtained from Euroimmun Myositis Profile 3 with EUROBlot Master 44, EUROLine Scanner and software expressed as density units. Reference range 0-10 density units.

Supplemental Figure S1. *Amino acid sequence alignment of human KARS and IARS proteins*

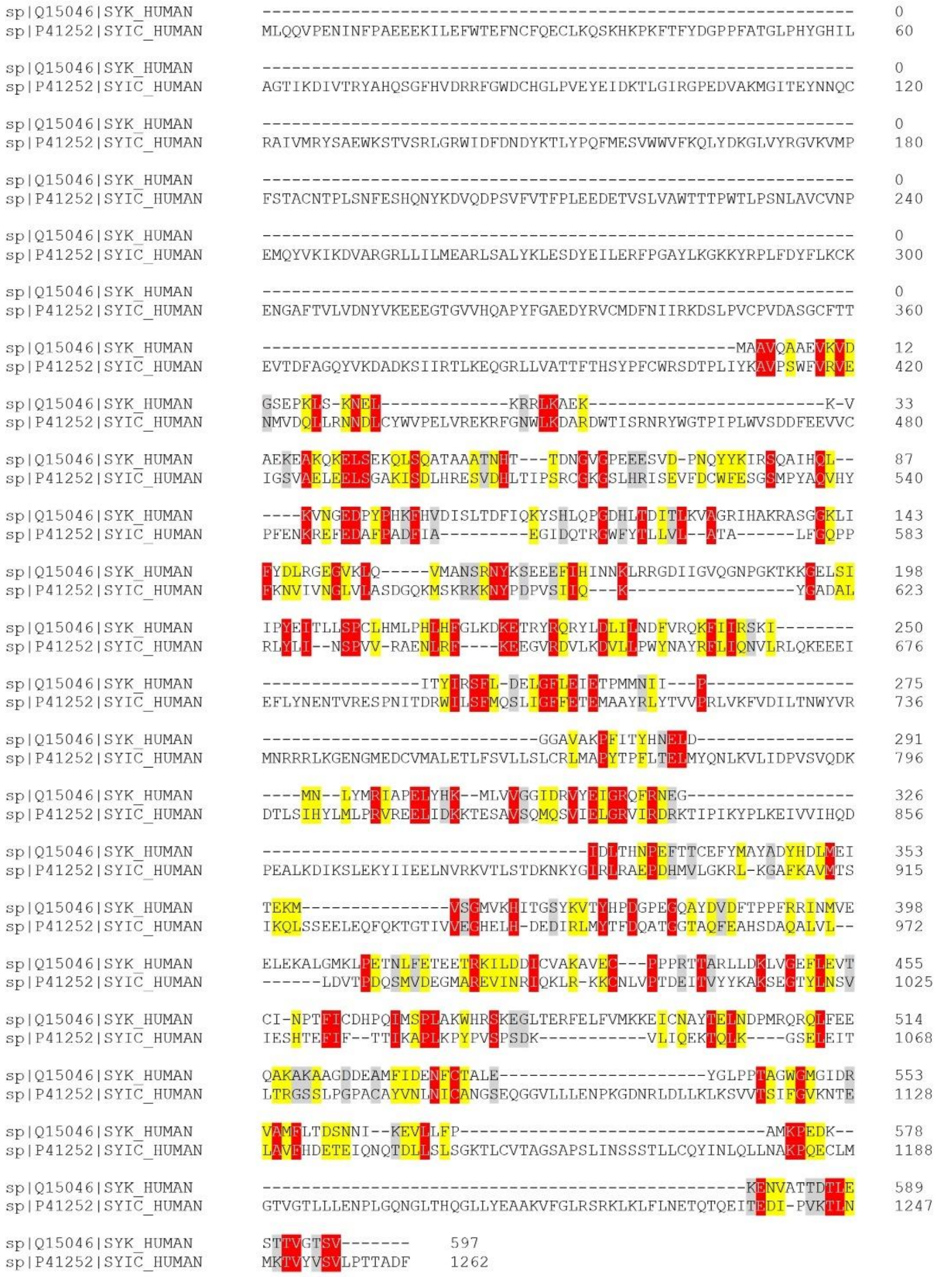

Legend: Multiple alignment was carried out by Clustal Omega (RRID:SCR_001591). Red background with white letters shows strictly conserved residues; yellow background indicates conservative substitutions; grey background shows semi-conservative substitutions. Gaps are indicated as dashes. Percent identity and similarity of human KARS to IARS are 8.61% and 15.73% respectively.

Supplement Figure S2: Correlations of HEp-2 IIF Staining Patterns with Levels of Anti-IARS and Anti-KARS Autoantibodies Measured by PMAT

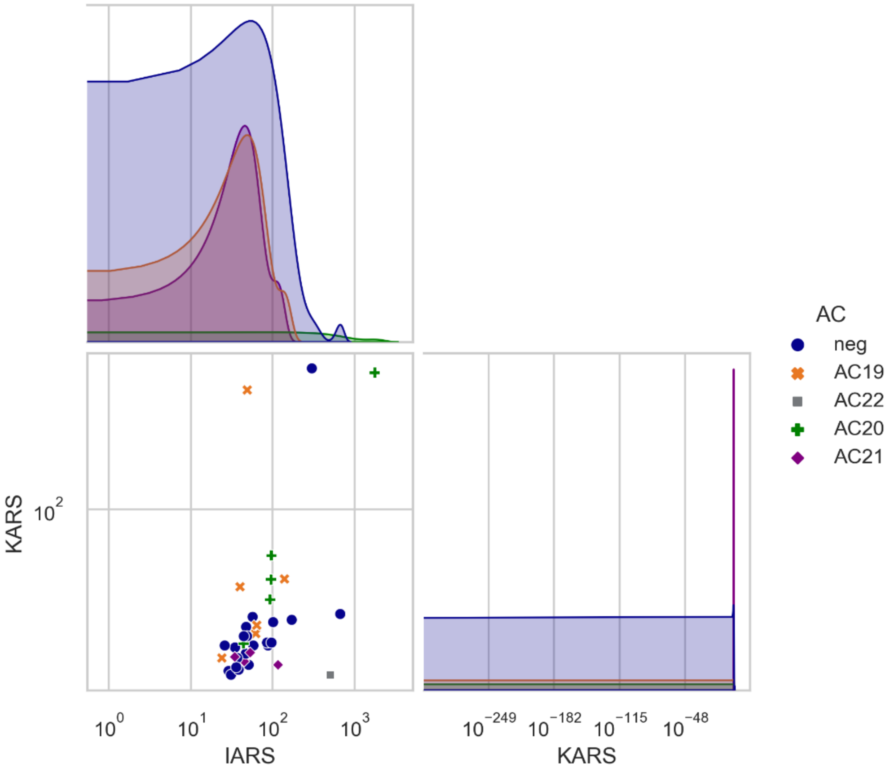

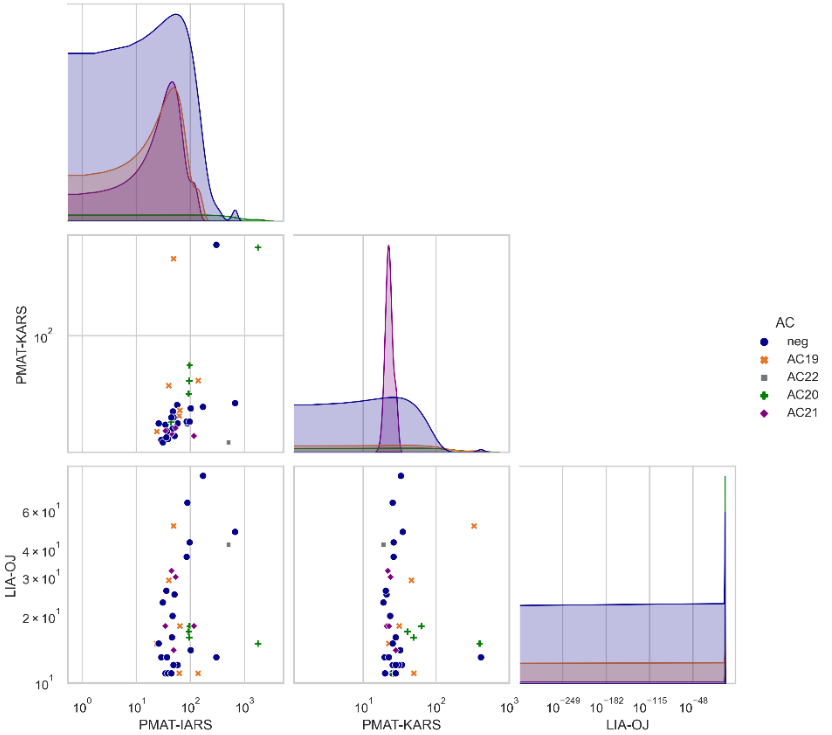

Supplement: Supplementary file 1 [file diagnostics-13-00156-s001.zip › diagnostics-2043719-supplementary.pdf]
